# Overcoming Barriers in the Implementation of Programs for Breast and Cervical Cancers in Cali, Colombia: A Pilot Model

**DOI:** 10.1200/JGO.19.00054

**Published:** 2019-07-01

**Authors:** Armando Sardi, Mavalynne Orozco-Urdaneta, Carolina Velez-Mejia, Andres H. Perez-Bustos, Carlos Munoz-Zuluaga, Farah El-Sharkawy, Luis Gabriel Parra-Lara, Patricia Córdoba, David Gallo, Michelle Sittig, Mary Caitlin King, Carol Nieroda, Katherin Zambrano-Vera, John Singer

**Affiliations:** ^1^ Institute for Cancer Care, Mercy Medical Center, Baltimore, MD; ^2^ Partners for Cancer Care and Prevention, Baltimore, MD; ^3^ Fundación para la Prevención y Tratamiento del Cáncer, Cali, Colombia

## Abstract

Breast and cervical cancers are leading causes of mortality among women in Latin America. Colombia has universal health care and a government-sponsored 10-year cancer control plan focused on prevention, early detection, and treatment. However, many administrative and social barriers have hindered its success, and a majority of patients are diagnosed at a late stage. Established in 2012, Partners for Cancer Care and Prevention (PFCCAP) works to decrease the burden of these cancers by mitigating the obstacles women face during their cancer diagnosis and treatment. Through community outreach meetings with medical personnel, hospital directors, and government officials, PFCCAP identified major barriers, including lack of trained health care personnel, few centers with adequate screening equipment, and a fragmented health system with significant administrative delays and poor continuity of care. Its solution included monthly teleconferences, biannual on-site training, quality control programs, and improved access to screening equipment. PFCCAP also initiated a patient navigation project. After implementation of the PFCCAP plan of action, from 2012 to 2018, the average time from initial consult to biopsy decreased from 65 to 20 days; from biopsy to diagnosis, 33 to 4 days; and from diagnosis to surgery, 121 to 60 days. To date, more than 1,500 women have benefited from this initiative, which has expanded to other regions. Overall, PFCCAP is creating centers of excellence in strategically located hospitals and promoting the implementation of national guidelines. Although several barriers still exist, PFCCAP is helping to implement an efficient health care model that can be replicated in other underserved populations.

## INTRODUCTION

Breast and cervical cancers are leading causes of morbidity and mortality in Latin America, partially as a consequence of the social and economic inequalities in most of these countries.^1,2^ According to the Colombian population registry, 61.7% of breast cancer cases are diagnosed in advanced and/or metastatic stages.^3,4^ The Colombian government provides universal health care coverage, as legally mandated; however, it faces the challenge of controlling communicable and noncommunicable diseases.^5^ Now, more resources are allocated to controlling the most prevalent infectious diseases. However, with increasing attention on the morbidity and mortality of detectable and treatable breast and cervical cancers, prioritizing and redistributing resources become significant challenges.

In an attempt to control the cancer burden, in 2012, the government created a 10-year cancer control plan that aims to address risk, early detection, treatment, rehabilitation, and palliation. This strategy was formulated within the National Development Plan, which seeks to reduce health inequity. However, many barriers have hindered its implementation, and instead of a comprehensive cancer care program, Colombia has seen the disparities in access and treatment widen over time.^6-8^

Partners for Cancer Care and Prevention (PFCCAP) and its sister organization in Colombia, Fundación para la Prevención y Tratamiento del Cáncer, are 501(c)(3) nonprofit organizations established in 2012 to work toward the unification of the health system by helping to implement the governmental 10-year cancer control plan. In this report, the work of PFCCAP refers to the work performed by the two organizations, which are integrally connected. Their vision and mission are based on the concept that early detection saves lives and focused on decreasing the individual and community burdens of breast and cervical cancers by mitigating the obstacles women face during cancer diagnosis and treatment. Over the last 6 years, PFCCAP has identified important barriers to health care access and subsequently addressed these issues with a phased approach.^9^ Here we present the initial pilot results.

## METHODS

Cali, the capital city of the state of Valle del Cauca, is considered the third most important city in Colombia because of the number of citizens, thriving metropolitan area, economic and industrial statuses, and prime location along the Pacific coast. Currently, Cali is divided into five main health care networks, known as Empresas Sociales del Estado (ESEs), which provide coverage to 2,319,655 residents: Red de Salud Ladera, Red de Salud del Norte, Red de Salud del Centro, Red de Salud del Suroriente, and Red de Salud Oriente ESEs.

PFCCAP partnered with three of these health care centers that serve subsidized populations. Red de Salud Ladera ESE provides services for all urban and rural areas in the Andes Mountains of the Cali region, covering a total population of approximately 750,000. Red de Salud del Norte ESE reaches roughly 530,000 people and already has the basic infrastructure necessary to develop the breast cancer program. Finally, Red de Salud del Centro ESE is equidistant from the previous two institutions and has high-quality laboratory equipment necessary for cervical cancer screening and the potential to support the breast cancer program. One key advantage of these health care centers is the presence of a stable functioning health system with strong promotion and prevention activities, such as young adult and mother-child programs, already in place. Although this was the initial implementation of a cancer program, the required infrastructure to provide patient care and community outreach was already established. As the pioneer of this initiative, PFCCAP aims to support these institutions with the implementation of a comprehensive cancer program. After program establishment (approximately 3 to 5 years), PFCCAP will play a limited role, because these facilities should become self-sustainable and require oversight only for quality control.

The understanding of the Colombian health system by leaders of PFCCAP was obtained through the following: author A.S. was born in Cali, trained as a Colombian physician with additional training in the United States as a general surgeon and surgical oncologist, and is currently a leader in cancer treatment in the United States and president of PFCCAP; from 2006 to 2012, large groups of medical personnel from different specialties participated in on-site medical missions at all levels of medical care facilities in Cali, during which time more than 35,000 medical visits and 3,500 surgical procedures were performed, information was gathered and analyzed, and ultimately the mission and vision of organizations were changed; government meetings were held at the local, regional, and national levels, including the Ministry of Health and the National Cancer Institute (NCI) of Colombia; meetings were held with directors and administrators of health maintenance organizations (HMOs); and quality control guidance was provided via on-site visits and training in various specialties, including radiology, pathology, general surgery, surgical oncology, gynecology oncology, nursing, and social work.

In Colombia, health care is categorized by level of care in ascending order. Level 1 institutions provide nonhospital basic care. Level 2 institutions provide level 1 services plus intermediate care, including some diagnostic testing and basic operating rooms. Level 3 institutions offer specialized care, because they are technologically advanced, and maintain services included in level 1 and 2 institutions. Insurers (HMOs) and health centers (levels 1, 2, and 3) must be contracted to guarantee community health care services. The insured are divided into two regimes (contributory and subsidized) on the basis of individual financial status, as legally mandated. If an individual is unable to pay for health care, enrollment is required in the subsidized government program.

We hypothesize that a coordinated program of screening and early diagnosis of breast and cervical cancers can be achieved using a stepwise approach and by transforming primary care hospitals into centers of excellence. These centers will be easily accessible to the community, provide rapid diagnoses and expedited referrals, and therefore improve diagnosis time and decrease delays and treatment-associated costs.

## RESULTS

### Barriers

On the basis of multifaceted comprehensive data from personal experience, meetings and discussions, and participation in medical missions, the following barriers to establishing a comprehensive cancer program were identified:

#### Fragmented administrative health care system.

The most significant barrier identified was a fragmented health care system with multiple administrative delays. Patients have difficulty accessing screening programs and obtaining authorizations for services covered by the governmental 10-year cancer control plan. Additionally, there is limited or nonexistent communication between hospitals, especially between level 1 to 2 and level 3 facilities. Despite the universal coverage, HMOs have absolute control in determining authorizations and require a lengthy regulatory administrative process before patients receive treatment. In 2012, calculations were based on patient medical record review and a survey of 105 women regarding time between each treatment or procedure. The average waiting time for a patient with breast cancer from initial consult to biopsy was 65 days (range, 40 to 90 days); from biopsy to diagnosis, 33 days (range, 15 to 60 days); and from diagnosis to surgery, 121 days (range, 60 to 165 days). Another example of administrative delay is the authorization of chemotherapy treatments. They are often dose approved by cycle versus entire chemotherapy regimen, and treatments occur at different health facilities.

#### Limited number of centers and scarcity of equipment required for early diagnosis.

On the basis of federal regulations of hospital services, patients must be transferred to facilities in ascending order, which contributes to treatment delays. The equipment in level 1 and 2 institutions varies widely, with some facilities having considerably more technology than others. For example, some may have electronic medical records, whereas others maintain handwritten records. Another variation across institutions is in regard to the Breast Imaging Reporting and Data System. In lower levels of care, the incidence of a Breast Imaging Reporting and Data System score of 0 (inconclusive imaging) can be up to 23%, significantly higher than the expected standard of < 10%. This leads to additional tests at higher-level institutions and causes additional interruptions in care. The lack of equipment and knowledge in level 1 to 2 hospitals and resulting oversaturation of level 3 centers further delay diagnosis. In addition, long distances and the scarcity of level 3 hospitals create challenges in getting to the correct centers expeditiously.

After several years of evaluating the health system, it was evident to PFCCAP that the concept of screening is not well understood and/or applied to breast and cervical cancers. Numerous administrative barriers and referral delays have greatly weakened these programs. Sadly, these same barriers can be applied to the diagnosis and treatment of other cancers in Colombia.

#### Socioeconomic barriers.

The lower socioeconomic status of the Colombian population contributes to and exacerbates health care problems. Wide variation in development and access to health care facilities within a region, social stigmas, misconceptions, and a general lack of awareness of breast and cervical cancers exist in South America.^10^ Common misconceptions include that cancer is contagious, is infectious, causes pain with physical contact, or requires sexual abstinence. These misconceptions often lead to abandonment by male partners when women are diagnosed with cancer. Additionally, socioeconomic barriers often negatively impact patients seeking medical care. For example, housing and food expenses often consume the entirety of the family income, leaving little or no financial means for medical costs, such as transportation. Finally, in many instances, patients are required to deliver biopsy samples to the pathology laboratory themselves, leading to issues with sample preservation and loss of specimens, without quality control. This is also the case for medical records.

#### Deficit in the number of trained personnel at primary health care centers.

There is a lack of adequately trained personnel at the centers involved in cancer care, mainly because of a limited awareness of the 10-year cancer control plan among practicing physicians, nurses, and administrators. When a primary care physician orders a biannual screening mammogram (standard for women age ≥ 50 years), an HMO may deny authorization, and the patient must return to the primary care physician for gynecology referral. Furthermore, when breast biopsy results confirm carcinoma, additional orders are necessary to perform testing, such as hormone receptor status. Therefore, when the patient meets with the medical oncologist, incomplete pathologic information further delays initiation of therapy, resulting in inappropriate cancer care.

This problem stems from insufficient oncology education in medical schools and limited training of oncology physicians by the NCI of Colombia. Annually, the Colombian NCI trains two surgical oncologists, two breast surgeons, four gynecologist oncologists, and two medical oncologists. Several universities in Colombia are beginning to offer medical oncology fellowships; however, they are not yet available in surgical oncology.

### Solutions

After identifying these major barriers, PFCCAP proposed realistic targeted solutions to address these problems (Table 1). Over the course of 7 years (2012 to 2018), PFCCAP received support from the Susan G. Komen Foundation, and through the implementation and funding of the PFCCAP plan of action the following results have been achieved:

**TABLE 1:**
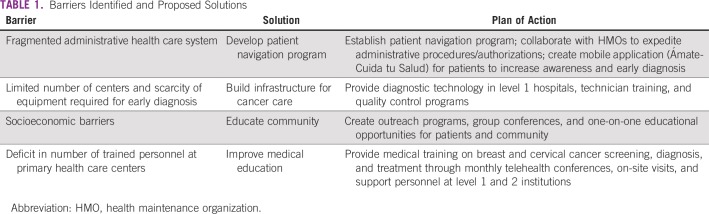
Barriers Identified and Proposed Solutions

#### Patient navigation program.

PFCCAP worked to create a patient navigation program that acts as a bridge between the patient and health care system. The purpose is to integrate the fragmented health care system by coordinating services, expediting administrative procedures, and providing guidance for patients during cancer diagnosis and treatment.

Patient navigators are professionals who are trained in health care or health-associated fields, such as nurses, medical assistants, psychologists, and social workers. The director of each institution must authorize the navigation program, and the navigator is required to obtain affiliation with a health care provider or insurer. The unique and specialized training of the navigator includes effective communication skills, networking abilities, and a universal understanding of the Colombian health system.

**FIG 1:**
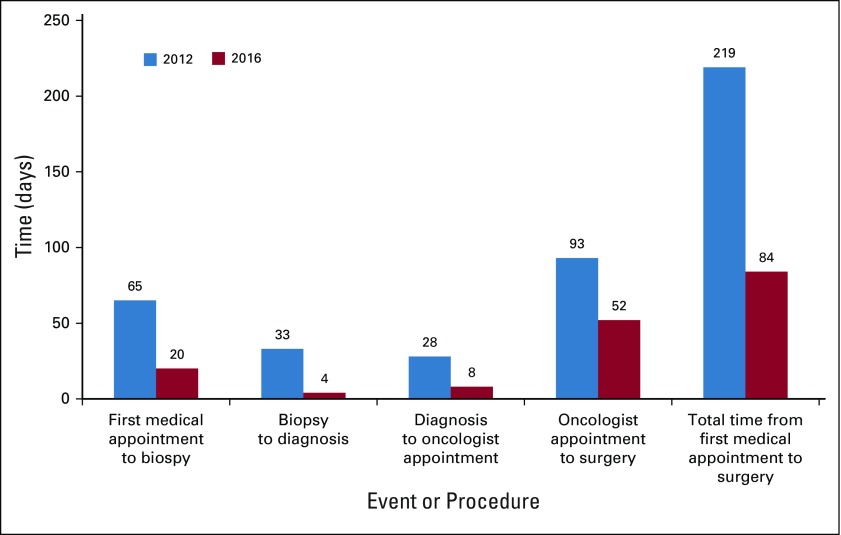
Results of integrating the Colombian health system. Comparison of wait times for patients with breast cancer in 2012 (n = 105) versus 2016 (n = 9).

PFCCAP hired and trained administrators as navigators and established the program in strategically located hospitals for cancer care coordination. In 2016, after the initiation of the patient navigation program, nine patients with breast cancer successfully navigated through the system with decreases in patient wait time (Fig 1) and number of patients lost to follow-up, demonstrating the impact of this initiative. The mean time from initial consult to biopsy decreased from 65 to 20 days; from biopsy to diagnosis, 33 to 4 days; and from diagnosis to oncologist appointment, 28 to 8 days. Overall, the total time from the first medical appointment to surgery decreased from 219 to 84 days. This is similar to the 2012 data in regard to population and communities served.

The biggest navigation challenge has been that multiple authorizations are required for a single treatment, necessitating significant effort to navigate a single patient. Frequently, a protective action is required to make sure that the HMO follows the recommended guidelines stated in the 10-year cancer control plan. From 2012 to 2018, 108 legal actions were initiated with the help of PFCCAP to obtain appropriate treatment for patients with breast and/or cervical cancer.

Currently, PFCCAP is also in the process of developing a mobile application called Ámate-Cuida tu Salud as a complement to the navigation program. This mobile application aims to provide basic education on breast and cervical cancers, identify patients at risk, and navigate patients more efficiently. The rationale is that smartphones have high usage among all socioeconomic strata and can potentially reach patients who would not usually seek medical attention or those located in rural communities. The results of the evaluation phase were presented at the 2018 Breast Health Global Initiative Global Summit in Seattle, Washington.^11^ This initiative was evaluated from August 2017 to May 2018, with the Ámate app advertised in the waiting room of the Cali hospital. A total of 830 women downloaded and used the app, which identified 131 users (16%) at risk for breast and/or cervical cancer eligible for the national screening program. Thirty-two users (24%) were successfully screened. Specific barriers to enrollment included unwillingness to be enrolled, limited available appointments at health care centers, and access denial as a result of out-of-network health care coverage. We found that the mobile app is a low-cost and accessible tool that identifies women at risk for breast and cervical cancers and detects barriers to early cancer detection. This innovative approach has the potential to reach rural areas and can be replicated in other underserved countries.

#### Building local capacity for cancer care.

Providing technology for early diagnosis in strategically located hospitals in Cali improves access to care for underserved populations. PFCCAP provided funds for a digital mammography machine, which performs 3,036 mammograms per year, and two video colposcopes, which yield 1,500 uterocervical cytologies per year. Along with this, a program for technician training and a quality control system were initiated. With the support necessary to implement the governmental 10-year cancer control plan, some of these hospitals are now making important progress toward becoming centers of excellence. However, political and administrative changes have limited the pace of implementation and made maintaining adequate personnel in some of these institutions challenging. Nonetheless, some HMOs have started using electronic medical records and computerized approval for certain procedures, which expedite the authorization process and tremendously alleviate the inefficient pathway patients were previously required to follow.

#### Community education.

Demystifying breast and cervical cancers is a challenge. By providing patient education and creating lasting educational programs, many misconceptions and stigmas held by both women and men have decreased. Group conferences, one-on-one education, support groups, and outreach programs have benefited more than 1,500 patients and their families. Focusing on creating a comprehensive cancer program, PFCCAP also established a project to improve the quality of life and self-image of women undergoing chemotherapy and/or mastectomy, which has provided 592 breast prostheses, 592 mastectomy bras, and 422 wigs to date.

#### Improving medical education.

PFCCAP started a medical training program directed toward primary care physicians, nurses, physical therapists, technologists, general surgeons, radiologists, and medical students. Topics, including risk factors, screening, diagnosis, and treatment of breast and cervical cancers, are taught through monthly telehealth conferences and on-site visits at least twice a year. This initiative was initially funded in 2012 by a 1-year grant from the Conquer Cancer Foundation as part of the International Innovation Grant for cervical cancer; however, PFCCAP expanded the program to include breast cancer, and it remains active today. Self-assessment questions are given at the beginning and end of each session. After the live lectures, conferences are available online, allowing access to many. To date, 598 health professionals have been trained, and the continuing medical education program includes three high-volume institutions.

#### Program expansion.

In 2016, the program expanded to the city of Bucaramanga, Colombia, in the region of Piedecuesta. This is a smaller community, with a homogeneous population that has a stable political administration. The barriers encountered in this region are similar to those seen in Cali, although with some idiosyncrasies in each region. However, all of the projects included in the plan of action are being successfully implemented. In addition, a human papillomavirus vaccine education program dictated by physicians and directed to public school principals and students has been created (results to be reported separately).

## DISCUSSION

Breast and cervical cancers carry the highest rates of cancer-related mortality for women in Colombia, representing 15.3% (2,815 patients) and 11.5% (2,116 patients) of cancer-related deaths per year, respectively.^12^ Age-adjusted incidence rates (reported as per 100,000 women per year) vary according to the six population-based cancer registries of the country.^13-15^ Breast cancer rates range from 26.3 to 81.3 patient cases, whereas cervical cancer rates range from 8.5 to 26.8 patient cases. Likewise, deaths have been reported as between 12.9 and 30.9 for breast cancer and 4.9 and 26.3 for cervical cancer^16-19^ (Table 2). This is especially true for less educated women and women of lower socioeconomic status, who have a higher risk of dying as a result of breast or cervical cancer.^6,21-23^ This population faces a higher prevalence of risk factors, such as smoking, alcohol abuse, obesity, occupational exposure, housing circumstances, lack of awareness, distance from health care facilities, and significant economic burden of taking time off from work for appointments. This ultimately translates into lower health care use and greater inequality.^8^

**TABLE 2:**
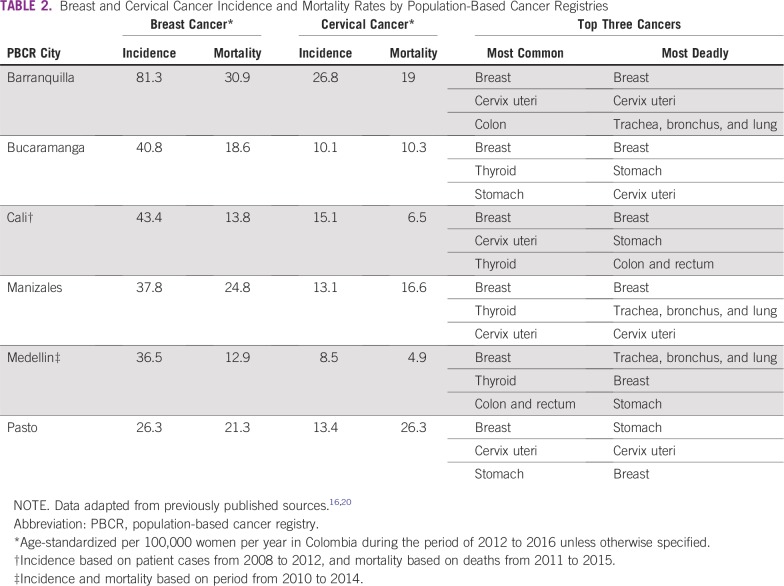
Breast and Cervical Cancer Incidence and Mortality Rates by Population-Based Cancer Registries

It is important to recognize that the health system in Colombia has multiple advantages. It works well for the diagnosis and treatment of other diseases, and a majority of patients have health insurance (94.73%), even if unemployed or homeless. There are significant entities, governmental and nonprofit, interested in improving cancer care with local and national government participation. Hence, multiple projects to improve the health of citizens, such as mother-child and young adult programs, have been successfully implemented.

In Cali, a limited network of care with social workers and electronic medical services exists, making it easier to contact patients, provide follow-up and additional health care services, and educate the community. Additionally, the government has a 10-year cancer control plan (2012 to 2021) that aims to decrease the prevalence of modifiable cancer risk factors, decrease rates of death resulting from cancer through early detection, and improve quality of care and quality of life for patients and survivors^24^ (Table 3). Although the plan has been clearly outlined in six strategic lines, each with defined goals and actions at the political, community, and services levels, it has only been partially implemented.

**TABLE 3:**
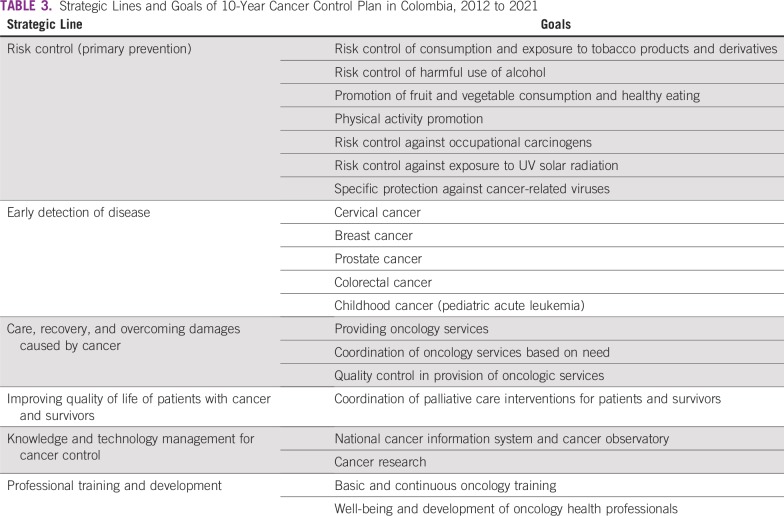
Strategic Lines and Goals of 10-Year Cancer Control Plan in Colombia, 2012 to 2021

PFCCAP, as well as previous investigators, has shown that the barriers to implementation of the 10-year cancer control plan are multifactorial.^8,22,25^ Although other initiatives are trying to improve the health system, PFCCAP is more focused on implementation through a well-coordinated stepwise approach and by connecting key stakeholders to ensure patients are not only diagnosed but also treated. First, it is important to recognize that simultaneous implementation across the country for all types of cancer is not only impractical but also impossible because of differences in education, economic status, and services available in each area. Second, it is important to identify the most prevalent cancer in each region and define realistic goals that can be achieved within a specific timeframe. PFCCAP is focused on breast and cervical cancers because of the high incidence and mortality of these cancers among Colombian women.

Partnering with strategically located health care facilities that are easily accessible to the population in need can improve patient circumstances and health care use. In order for these health care facilities to become centers of excellence, a team consisting of trained administrators, primary care physicians, radiologists, nurses, and navigators is needed. Each center must understand the benefits of an integrated cancer care program and be capable of screening women, establishing early diagnoses, and providing rapid referrals to level 3 hospitals for multidisciplinary treatment. The support of a navigation program ensures proper referral, follow-up, and evaluation of outcomes for quality control. Although the administrative organization can be shared with other health care programs for noncommunicable diseases, the personnel involved with treatment of patients with cancer requires special training, because there are unique issues to consider. Additionally, even though some services can be shared between different types of cancer, it is important to recognize that each type of cancer is not a single disease; rather, cancer comprises a variety of conditions that are treated differently and require specialized services, equipment, and interdisciplinary collaboration. Therefore, when implementing these programs, additional guidance is needed to avoid overwhelming health care personnel and further hindering the implementation process. Also, a primary care physician who is trained in the management of the breast and/or cervical cancer program is important for supervising the adequate functioning of the 10-year cancer control plan. Finally, a continued quality control system is required to maintain proficiency in the services provided.

Breaking the status quo will require a variety of foundations and interested parties working together. Because of political interests, cooperation between the five different health care networks in Cali is fragmented and limited. Collaboration will allow the sharing of resources, facilitate patient access to care, and decrease costs. The problem is too vast to be solved by one person, hospital, or the government alone. A shielded resolution to protect programs or initiatives that demonstrate quality care must be created by the government, so changes in political administrations do not eradicate them. Also, HMOs have immense unrestrictive power in determining necessary care, with limited oversight by the government. Increasing the number of patients diagnosed, but not providing the treatment or palliation needed, worsens the problem and creates a significant psychological burden for those diagnosed. Currently, the governmental tertiary care hospitals are a major barrier, because they have considerable economic needs. Therefore, participation of the private industry and private level 3 hospitals is paramount to bridge the gap. Unless key stakeholders work together, late cancer diagnoses and fragmented care will continue.

In 2018, the NCI of Colombia reported that the country had only 25 certified locations offering joint chemotherapy, radiotherapy, and surgical services.^13^ This exposes the lack of infrastructure for cancer care and the administrative and logistic inconveniences patients face in obtaining appropriate treatment. Adding to the problem, a low-resource setting that has high turnover of personnel makes it difficult to comply with the established quality standards.^15,26^ Training in cancer care needs to improve at the university level, and a well-defined curriculum with a guided program should be implemented. Furthermore, the country needs to expand the fellowship training program in oncology. However, as the physician who can identify patients in need of screening or who are at high risk for cancer, the primary care physician can make substantial changes in time to diagnosis and needs to become an integral part of the cancer support system.

PFCCAP was able to identify several obstacles in health care. Targeted solutions and action plans improved time to referral and diagnosis for women with breast or cervical cancer. By creating centers of excellence in strategically located hospitals within partnered health care networks, the cancer program was effectively initiated in underserved communities. Although some obstacles are still present, a majority are administrative in nature. PFCCAP designed an intervention that efficiently addresses multiple limitations and allows the implementation of national guidelines.

Integrating the health care system and overcoming barriers are improving early diagnosis and treatment, leading to decreased morbidity and mortality among Colombian women. Restructuring of the current referral pattern for cancer management is necessary. Creating centers of excellence in strategically located level 1 and 2 hospitals will help implement the current governmental 10-year cancer control plan. Subsequently, level 1 and 2 hospitals will have the infrastructure to provide initial diagnosis and follow-up for patients with cancer, allowing level 3 hospitals to provide timely treatment. This reorganization saves time and resources while coordinating diagnosis and treatment. Although several barriers still need to be addressed, PFCCAP is helping to implement an efficient health care model that could be replicated in other underserved countries facing similar barriers.
